# Fatores de Risco Cardiovascular em Cardiologistas Especialistas pela Sociedade Brasileira de Cardiologia

**DOI:** 10.36660/abc.20200125

**Published:** 2021-04-08

**Authors:** Maria Emília Figueiredo Teixeira, Priscila Valverde de O. Vitorino, Celso Amodeo, Tânia Martinez, Andréa Araujo Brandão, Eduardo Costa Duarte Barbosa, Audes Diógenes Magalhães Feitosa, Paulo Cesar B. Veiga Jardim, Ana Luiza Lima Souza, Weimar Kunz Sebba Barroso

**Affiliations:** 1 Universidade Federal de Goiás Liga de Hipertensão Arterial GoiâniaGO Brasil Universidade Federal de Goiás - Liga de Hipertensão Arterial, Goiânia, GO – Brasil.; 2 Universidade Federal de Goiás Programa de Pós-Graduação em Ciências da Saúde GoiâniaGO Brasil Universidade Federal de Goiás - Programa de Pós-Graduação em Ciências da Saúde, Goiânia, GO - Brasil.; 3 Pontifícia Universidade Católica de Goiás Escola de Ciências Sociais e da Saúde GoiâniaGoiás Brasil Pontifícia Universidade Católica de Goiás - Escola de Ciências Sociais e da Saúde, Goiânia, Goiás - Brasil.; 4 Universidade Federal de São Paulo Escola paulista de Medicina São PauloSP Brasil Escola paulista de Medicina da Universidade Federal de São Paulo (Unifesp EPM), São Paulo, SP - Brasil.; 5 Hospital do Coração São PauloSP Brasil Hospital do Coração (HCor) – Lipids, São Paulo, SP - Brasil.; 6 Universidade do Estado do Rio de Janeiro Rio de JaneiroRJ Brasil Universidade do Estado do Rio de Janeiro (UERJ) – Cardiologia, Rio de Janeiro, RJ - Brasil.; 7 Instituto de Cardiologia Laboratório de Investigação Clínica Porto AlegreRS Brasil Instituto de Cardiologia - Laboratório de Investigação Clínica (LIC), Porto Alegre, RS - Brasil.; 8 Universidade Federal de Pernambuco Laboratório de Imunopatologia Keizo Asami RecifePE Brasil Laboratório de Imunopatologia Keizo Asami - Universidade Federal de Pernambuco - Clínica Médica, Recife, PE - Brasil.; 9 Universidade Federal de Goiás GoiâniaGO Brasil Universidade Federal de Goiás – Cardiologia, Goiânia, GO – Brasil.

**Keywords:** Doenças Cardiovasculares, Cardiologistas, Fatores de Risco, Antropometria, Hipertensão, Dislipidemias, Diabetes Mellitus, Estilo de Vida

## Abstract

**Fundamento:**

Principal causa de morte em todo o mundo, as doenças cardiovasculares (DCV) e sua prevalência nos médicos cardiologistas são pouco conhecidas.

**Objetivos:**

Descrever os hábitos de vida e os fatores de risco cardiovascular e verificar a prevalência de diagnóstico, conhecimento e controle dos fatores de risco cardiovasculares (FRCV) de médicos cardiologistas associados e especialistas pela Sociedade Brasileira de Cardiologia.

**Métodos:**

Estudo multicêntrico nacional transversal que avaliou cardiologistas brasileiros por meio de questionário sobre hábitos de vida, doenças preexistentes, medicações em uso, medidas antropométricas, pressão arterial e dosagens de glicose e lípideos sanguíneos.

**Resultados:**

Foram avaliados 555 cardiologistas, 67,9% do sexo masculino, média de idade de 47,2±11,7 anos. A maioria era não tabagista (88,7%), fisicamente ativa (77,1%), consumia bebida alcóolica (78,2%), com circunferência abdominal normal (51,7%) e excesso de peso (56,1%). As prevalências de hipertensão arterial sistêmica (HAS), diabetes mellitus (DM) e dislipidemia (DLP) foram de 32,4%, 5,9% e 49,7%, respectivamente e, destes, apenas 57,2%, 45,5% e 49,6% sabiam ter as doenças.

**Conclusões:**

Os cardiologistas brasileiros participantes do estudo apresentaram prevalências significativas de HAS, DM e DLP, mas apenas a metade dos participantes sabia ser portador dessas condições e, entre eles, as taxas de controle eram baixas para HAS e DLP, apesar de os cardiologistas serem profissionais detentores de conhecimento diferenciado sobre esses FRCV. Os achados representam um alerta para a abordagem dos FRCV em cardiologistas brasileiros e estimulam a realização de estudos futuros.

## Introdução

Dentre os fatores de risco cardiovascular (FRCV), aqueles com maior impacto no aumento das taxas de morbidade e mortalidade são hipertensão arterial sistêmica (HAS), diabetes mellitus (DM) dislipidemia (DLP) e tabagismo.^1^ Além disso, os hábitos de vida desfavoráveis levam ao excesso de peso e, juntos, interferem de maneira significativa na prevalência desses fatores,^2^ com consequente aumento da incidência de desfechos cardiovasculares, tais como morte súbita, acidente vascular encefálico (AVE), infarto agudo do miocárdio (IAM), insuficiência cardíaca, doença arterial periférica e doença renal crônica.^3^^–^^5^

Os profissionais de saúde, incluindo a classe médica,, especialmente o cardiologista, assumem papel fundamentalcomo responsáveis por diagnosticar e tratar doenças cardiovasculares.^6^ Além disso, o cardiologista brasileiro é visto, com frequência, como o responsável pelos cuidados com a saúde global do paciente adulto.^7^ Portanto, é de se esperar que, além de cuidar, os cardiologistas também sirvam de modelo a ser seguido e, principalmente, que assumam uma postura pessoal de hábitos de vida saudáveis.^8^

Poucos são os estudos que avaliaram o risco cardiovascular e os hábitos de vida de cardiologistas brasileiros;^9^ portanto, foram objetivos deste estudo: (1) verificar hábitos de vida e FRCV e (2) identificar as prevalências de diagnóstico, conhecimento e controle de HAS, DM e DLP em médicos cardiologistas associados e especialistas pela Sociedade Brasileira de Cardiologia (SBC).

## Métodos

### Tipo de estudo, população, amostra e critérios de inclusão

#### Estudo multicêntrico nacional transversal descritivo.

Em 2017, o Brasil possuía 451.777 médicos, com aproximadamente 25.000 (5,5%) cardiologistas;^10^ destes, 11.495 tinham o título de especialista em Cardiologia (TEC).^11^ A população de referência foi constituída por 14.201 médicos cardiologistas sócios da SBC em 2017, distribuídos em todo o território nacional, com sociedades estaduais em 24 unidades federativas. Optou-se por realizar a pesquisa com cardiologistas portadores do TEC/SBC para uniformização da amostra em relação ao nível de conhecimento científico.

A amostra foi de conveniência, tendo sido incluídos no estudo 555 médicos com o TEC/SBC e membros ativos da SBC, o que representa 4,8% da população de referência.

### Locais de realização e coordenação do estudo

Todos os 24 representantes regionais da SBC/Diretoria de Prevenção em Saúde Cardiovascular (FUNCOR) foram convidados a integrar o grupo de pesquisadores deste projeto. Desses, 15 aceitaram o convite e, juntamente com outros três centros convidados [Instituto Dante Pazzanese de Cardiologia (IDPC), Liga de Hipertensão Arterial da Universidade Federal de Goiás (LHA/UFG) e Unidade de Hipertensão da Universidade Estadual do Rio de Janeiro], totalizaram 18 centros de pesquisa que efetivamente integraram o grupo de investigadores e coinvestigadores que coletaram dados no período de maio até outubro de 2017.

A coleta de dados foi feita nas nos seguintes estados: Bahia, Distrito Federal, Goiás, Mato Grosso, Mato Grosso do Sul, Minas Gerais, Pará, Paraíba, Paraná, Pernambuco, Rio de Janeiro, Rio Grande do Norte, Rio Grande do Sul, Rondônia, São Paulo e Tocantins.

Todo o trabalho foi coordenado pela Diretoria da SBC/FUNCOR, juntamente com as instituições universitárias IDPC e LHA/UFG.

### Procedimentos do estudo

Foram realizadas reuniões presenciais com todos os investigadores em maio e junho de 2017 para discussão sobre o desenho do estudo e a coleta de dados. Cada investigador, após treinamento, capacitou sua equipe local para o seguimento rigoroso dos procedimentos do estudo. A coleta foi feita pelo próprio pesquisador médico responsável, ou por outros cardiologistas ou estudantes de medicina devidamente treinados.

Os participantes do estudo receberam explicações acerca do objetivo do estudo, forma de coleta dos dados avaliados e sobre o termo de consentimento livre e esclarecido (TCLE), que foi lido e assinado por todos antes do início de qualquer procedimento do estudo.

A entrevista foi realizada individualmente em ambiente privativo e em horário e local previamente acordados com os participantes. O formulário de entrevista continha perguntas referentes a dados pessoais, hábitos de vida e antecedentes pessoais de doenças. Também foram realizadas medidas antropométricas e de pressão arterial (PA) e exames de glicemia e perfil lipídico.

A idade foi calculada a partir da data de nascimento. O sexo foi categorizado em masculino e feminino. Os hábitos de vida avaliados foram tabagismo (sim/não); consumo de bebidas alcóolicas (não/sim, para qualquer quantidade de consumo) e prática de atividade física (sim/não e tempo semanal, sendo considerado ativo o praticante de pelo menos 150 minutos semanais).^12^

As variáveis antropométricas coletadas foram altura, peso e circunferência da cintura. A altura foi referida pelo participante;^13^ o peso foi aferido com utilização de balança digital OMRON HN-290T, após retirada de acessórios e calçados e com uso de roupas leves.^14^

O IMC foi calculado por meio da fórmula peso/altura^15^ e classificado em: baixo peso (< 18,5 kg/m^2^), eutrófico (18,5-24,9 kg/m^2^); sobrepreso (25-29,9 kg/m^2^); obesidade 1 (30-34,9 kg/m^2^), obesidade 2 (35 -39,9 kg/m^2^) e obesidade 3 (≥ 40 kg/m^2^).^16^

A circunferência da cintura foi medida com fita inextensível^14^ e considerada alterada se maior que 88 cm para mulheres e maior que 102 cm para homens.^17^

A aferição da PA foi realizada com esfigmomanômetro automático da marca OMRON, modelo HBP 1100.^18^^–^^20^ de acordo com a recomendação da 7ª Diretriz Brasileira de Hipertensão Arterial.^21^ Foram obtidas três aferições da PA, excluída a primeira medida e calculada a média das duas seguintes. Os participantes foram classificados a partir do valor da média pressórica em normotensos (PA ≤ 120/80 mmHg), pré-hipertensos (121-139/81-89 mmHg), ou hipertensos estágio 1 (140-159/90-99 mmHg), 2 (160-179/100-109 mmHg) ou 3 (PA ≥ 180/110 mmHg).^21^

A glicemia e os lipídeos séricos foram obtidos com os aparelhos On Call Plus e Mission Cholesterol, respectivamente. Todos os valores dos exames foram obtidos diretamente dos aparelhos em mg/dL, exceto o LDL, que foi calculado pela fórmula de Friedewald.^22^

Foram realizadas dosagens sem o jejum e, portanto, considerados os valores de glicemia alterados ≥ 160 mg/dL^23^ e portadores de DLP aqueles com LDL ≥ 130 mg/dL e/ou triglicérides ≥ 175 mg/dL.^24^

Para o diagnóstico de HAS, DM e DLP, foi considerado pelo menos um dos seguintes critérios: relato de ser portador, feito pelo próprio participante e/ou uso de anti-hipertensivos e/ou PA ≥ 140x90 mmHg na média das medidas casuais; uso de hipoglicemiantes orais e/ou de insulina e/ou glicemia capilar ocasional ≥ 200 mg/dL; uso de estatinas, fibratos, ezetimiba e/ou triglicérides ≥ 175 mg/dL e/ou LDL ≥ 130 mg/dL.

Foi considerado conhecimento da doença o relato do próprio médico sobre ser portador. Os dados referentes às frequências de HAS, DM e DLP foram analisados em relação aos obtidos na Pesquisa Nacional de Saúde (PNS)^25^ e no sistema de vigilância de fatores de risco para doenças crônicas não transmissíveis por inquérito telefônico (VIGITEL);^26^ para essa análise, foi considerado somente o autorrelato do participante (dados referidos).

Foram consideradas controladas a HAS com pressão arterial sistólica < 140 mmHg e pressão arterial diastólica < 90 mmHg, DM com glicemia < 200 mg/dL e DLP com LDL < 130 e triglicérides < 175 mg/dL.^21^^,^^23^^,^^24^

### Análise estatística

Os dados foram digitados no programa Excel para Mac versão 16.30 e analisados em *software* de análise estatística Stata, versão 14. Foi realizada estatística descritiva com apresentação de médias, desvio padrão e frequências absoluta e relativa.

### Aspectos éticos

O projeto, desenvolvido pela FUNCOR da SBC, gestão 2016/2017, foi aprovado pelo Comitê de Ética em Pesquisa (CEP) do IDPC, sob o número 2.016.859. Todos os participantes assinaram o TCLE antes de qualquer procedimento do estudo, que seguiu a Resolução 466/2012.

## Resultados

Foram avaliados 555 cardiologistas com idade média de 47,2±11,7 anos, sendo 159 (28,6%) da Região Centro-Oeste, 147 (26,5%) da Região Nordeste, 103 (18,6%) da Região Norte, 103 (18,6%) da Região Sudeste e 43 (7,7%) da Região Sul.

A maioria dos participantes da pesquisa era do sexo masculino, ativo, com tempo médio de atividade física de 200,0±106,8 minutos por semana, não fumante, e fazia uso de bebida alcóolica (Tabela 1).

**Tabela 1:** Descrição da amostra segundo o sexo, estilo de vida e condições gerais de saúde, n=555, 2017

Variável	n (%)
**Sexo**	
Feminino	178 (32,1)
Masculino	377 (67,9)
**Faixa étária**	
< 40 anos	183 (33,2)
≥ 40 anos	368 (66,8)
**Tabagismo**	
Sim	03 (0,5)
Não	492 (88,7)
Ex-tabagista	60 (10,8)
**Sedentarismo**	
Sim	127 (22,9)
Não	428 (77,1)
**Consumo de bebidas alcoólicas**	
Sim	434 (78,2)
Não	121 (21,8)
**Circunferência abdominal**	
Normal	285 (51,7)
Elevada	266 (48,3)
**Classificação segundo o índice de massa corporal**	
Sem excesso de peso	243 (43,9)
Sobrepeso	232 (41,9)
Obesidade	79 (14,2)

De acordo com as medidas obtidas durante a entrevista, a maioria dos médicos apresentou níveis pressóricos na categoria de pré-hipertensão e valores de glicemia, LDL e triglicérides dentro da normalidade (Tabela 2).

**Tabela 2:** Classificação dos cardiologistas de acordo com a medida da pressão arterial, glicemia casual e lipídeos séricos, 2017

Classificação	n (%)
**Pressão arterial (n=555)**	
Normotensos	204 (36,8)
Pré-hipertensos	264 (47,6)
Hipertensão I	75 (13,5)
Hipertensão II	08 (1,4)
Hipertensão III	04 (0,7)
**Glicemia casual (n=555)**	
Não alterada	548 (98,7)
Alterada	07 (1,3)
**LDL (n=538)**	
Não alterado	411 (76,4)
Elevado	127 (23,6)
**Triglicérides (n=547)**	
Não alterado	463 (84,6)
Elevado	84 (15,4)

**Figura 1:**
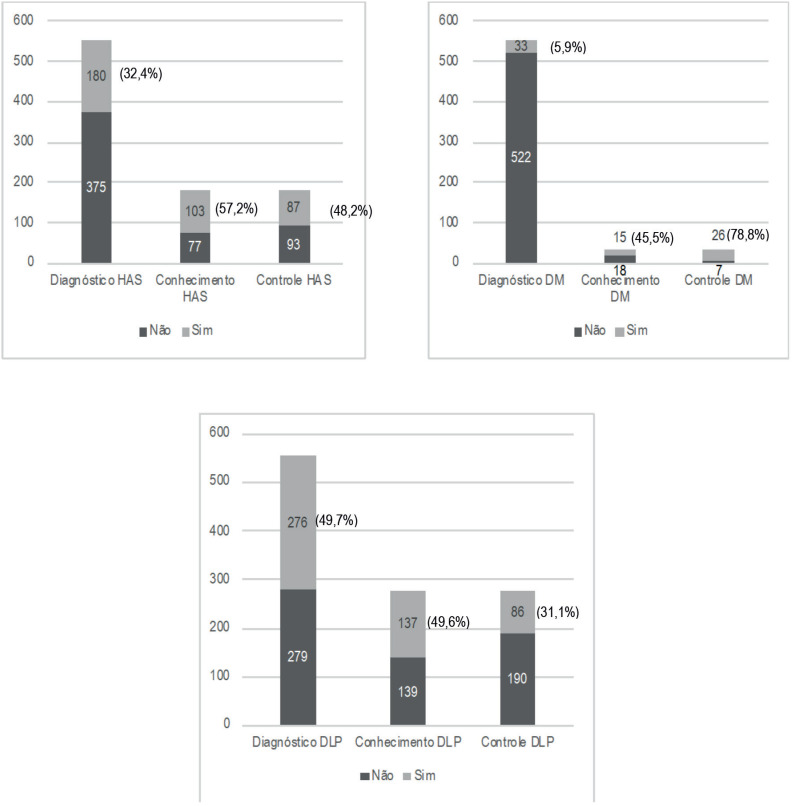
Prevalência de diagnóstico, conhecimento e controle de HAS, DM e DLP em cardiologistas, n=555, 2017. DLP: dislipidemia; DM: diabetes mellitus; HAS: hipertensão arterial sistêmica.

A prevalência de HAS foi de 32,4% (n=180); destes, 57,2% (n=103) conheciam essa condição, e 48,3% (n=87) estavam com a pressão controlada. A prevalência de DM foi de 5,9% (n=33); destes, 45,5% (n=15) sabiam que tinham a doença, e 78,8% (n=26) estavam com a glicemia dentro dos valores de normalidade. A dislipidemia apresentou valores de prevalência, conhecimento e controle de 49,7% (n=276), 49,6% (n=137) e 31,1% (n=86), respectivamente (Figura 1).

Em relação aos desfechos cardiovasculares, 4 (0,72%) cardiologistas referiram já ter apresentado IAM, e 1 (0,18%) referiu AVE. Todos os quatro portadores de doença arterial coronária diagnosticada estavam em uso de algum anti-agregante plaquetário.

A Tabela 3 apresenta as frequências dos FRCV e desfechos cardiovasculares do PNS,^25^ do VIGITEL^26^ e os achados do presente estudo, considerando somente as doenças que foram autorreferidas.

**Tabela 3:** Prevalência de fatores de risco e desfechos cardiovasculares na população geral e entre os cardiologistas. n = 555, 2017

	PNS	Vigitel	Cardiologistas (referido)	Cardiologistas (aferido)
Sedentarismo	46	61,9	22,9	-
Etilismo	24	17,9	78,2	-
Tabagismo	15	9,3	0,5	-
Hipertensão arterial	21,4	24,7	18,6	32,4
Diabetes mellitus	6,2	7,7	2,7	5,9
Dislipidemia	12,5	-	24,7	49,7
Infarto agudo do miocárdio	4,2	-	0,7	-
Acidente vascular encefálico	1,5	-	0,2	-

Fonte: PNS,^25^ Vigitel 2018^26^. PNS^:^ Pesquisa Nacional de Saúde.

## Discussão

Este é o primeiro estudo no Brasil a avaliar cardiologistas com TEC das cinco regiões geográficas para a presença de FRCV e hábitos de vida. Os cardiologistas apresentaram prevalências mais baixas de sedentarismo e tabagismo e mais altas de etilismo em relação a estudos que avaliaram a população em geral, como o PNS^25^ e o VIGITEL,^26^ e também prevalência maior de DLP, ligeiramente menor de HAS, e mais baixa de DM. Entretanto, as taxas de conhecimento de HAS, DM e DLP e as taxas de controle da HAS e DLP observadas foram baixas, considerando-se que a população estudada é de cardiologistas, conhecedores da importância do controle dos FRCV.

Na população brasileira, a prevalência de HAS varia de 30% a 36%;^27^^,^^28^ a de DM é 11,4%;^29^ e a DLP se divide em hipercolesterolemia, com prevalência de aproximadamente 45,5%,^30^ e hipertrigliceridemia, com prevalência na América Latina de 26,5% a 31,2%.^31^^,^^32^ Além disso, a prevalência de excesso de peso (sobrepeso/obesidade) no Brasil é de 57% em homens e 43% em mulheres.^33^ No grupo aqui estudado, considerando os dados referidos e aferidos, houve diagnóstico de 32,4% de HAS, 4,9% de DM, 51,7% de DLP (hipercolesterolemia e/ou hipertrigliceridemia), e 56% de excesso de peso (67,1% entre os homens e 32,2% entre as mulheres).

A falta de conhecimento de ser portador desses FRCV é sabidamente alta na população em geral, mas chama a atenção que também seja elevada entre os cardiologistas, levando-nos a considerar que haja uma negligência desses profissionais em relação aos cuidados com a própria saúde. Essa demora no conhecimento, diagnóstico precoce e tratamento adequado pode aumentar o risco de desfechos relacionados.^34^

Sabe-se que a educação em saúde para população leiga é capaz de gerar melhora dos hábitos de vida, impactando em redução das doenças cardiovasculares.^35^ Por esse motivo, surgiu o questionamento sobre a qualidade do autocuidado dos médicos cardiologistas, como portadores desse conhecimento específico. Estudantes de medicina avaliados para FRCV apresentaram prevalência semelhante à população geral de mesma faixa etária, exceto por maior sedentarismo e IMC, suscitando assim o debate sobre a carga horária elevada do curso, a qual pode influenciar na pequena disponibilidade de tempo para a prática de hábitos de vida saudáveis, em comparação com outros adultos jovens.^36^ Em outro grupo de estudantes de medicina, foram vistos níveis mais baixos de obesidade em comparação com a população da mesma idade, e níveis melhores de lipídeos séricos, mas um elevado consumo de *fast food* e bebidas alcoólicas, assim como maior sedentarismo, o que também pode ter como explicação a pequena disponibilidade de tempo e o elevado nível de estresse relacionado ao curso.^37^

Sabe-se também que, muitas vezes, a rotina de trabalho pode afetar negativamente a adoção de práticas de saúde e bem-estar, mesmo que o profissional seja detentor do conhecimento acerca do assunto, como são os profissionais da área de saúde.^38^ O trabalho nessa área exige a presença de equipes noturnas e, com frequência, esses profissionais trabalham em mais de um emprego. Dessa forma, dificilmente conseguem praticar exercícios físicos regularmente ou priorizar alimentos equilibrados do ponto de vista nutricional.

Por outro lado, a mesma discussão pode ser levantada sem a necessidade de enfatizar o trabalho noturno como malefício mais importante, mas considerando-se apenas a carga horária excessiva desses profissionais, independente do horário. Dois grupos diferentes avaliaram seus profissionais quanto à prevalência dos FRCV, incluindo toda a equipe multiprofissional na avaliação. Em um hospital geral, foi encontrada prevalência elevada de FRCV em todas as classes profissionais avaliadas.^39^ Resultados semelhantes foram encontrados em outro grupo, com uma situação ainda mais preocupante, que é a falta de conhecimento dessas pessoas acerca do seu quadro de saúde já alterado.^40^

Nos subgrupos de médicos cardiologistas *versus* não cardiologistas, não foram observadas diferenças significativas em relação aos níveis séricos de colesterol e frações, assim como em relação ao escore de risco de Framingham, mas os cardiologistas ingeriam mais bebidas alcoólicas, e ambos grupos estavam com IMC acima do ideal, em média.^41^

Em análise comparativa com os inquéritos populacionais PNS^25^ e VIGITEL,^26^ os cardiologistas avaliados no presente estudo referem menos tabagismo e sedentarismo, mas ingerem mais bebida alcoólica. Além disso, considerando apenas os FRCV referidos, relataram menos HAS e DM, porém mais DLP. Esses dados preocupam, não apenas pela falta de conhecimento, mas também porque colocam a questão da credibilidade de inquéritos que utilizam apenas dados referidos.

Sabe-se que HAS, DM e DLP^42^ resultam de fatores como genética e envelhecimento (não modificáveis), mas também têm relação com hábitos de vida e, nesse contexto, é de se esperar que indivíduos com melhor conhecimento em relação aos riscos cardiovasculares tenham hábitos mais saudáveis,^43^^–^^45^ Amplamente conhecedores do assunto, esperava-se que os médicos cardiologistas praticassem bons hábitos em sua totalidade, de modo a prevenir tais doenças, fato contradito em nossa amostra em relação ao consumo de bebidas alcoólicas, mas confirmado em relação ao tabagismo e à atividade física. Da mesma forma, encontramos prevalências semelhantes ou mesmo mais elevadas dos principais FRCV quando comparados à população, exceto em relação a DM.

Por fim, o percentual relatado de IAM (0,72%) e de AVE (0,18%) na amostra é bem menor que o da população em geral, o que pode estar relacionado ao uso regular e frequente de medicamentos, por conhecimento do tratamento adequado e facilidade do acesso a eles. Além disso, a idade média do grupo é baixa (47,2 anos) e pode justificar, em parte, a prevalência baixa dos desfechos IAM e AVE.^46^

O presente estudo apresenta como limitações: a ausência do HDL na avaliação de DLP, devido a uma limitação de análise do aparelho; não aplicação de instrumentos para avaliação de atividade física e do consumo de álcool, fato que pode ter superestimado essas taxas; e não foram obtidos exames de bioquímica em jejum. Vale ressaltar, contudo, que foram utilizados aparelhos iguais para aferições tanto de antropometria quanto da PA e da bioquímica sanguínea, com treinamento prévio dos coinvestigadores e coordenação geral dos centros de referência, demonstrando padronização adequada de procedimento.

Destacamos ainda que a amostra não foi representativa dos cardiologistas da SBC, pois se trata de uma amostra de conveniência, fato que pode relativizar os resultados e as discussões apresentadas. Entretanto, foram avaliados cardiologistas em todo o território nacional e, portanto, esse estudo representa um alerta para a abordagem das condições identificadas e para a realização de estudos futuros em cardiologistas brasileiros.

## Conclusão

A maioria dos cardiologistas era do sexo masculino, ativo, não fumante; fazia uso de bebida alcóolica; e apresentava prevalências significativas de HAS, DM e DLP, próximas às de outros levantamentos em populações brasileiras. Entretanto, embora tenham conhecimento sobre esses FRCV, aproximadamente a metade sabia ser portador dessas condições e estava com a pressão controlada; além disso, um terço estava com os lipídeos dentro dos valores de normalidade, mas a maioria estava com a glicemia controlada. Os achados representam alerta para adequada abordagem dos FRCV em cardiologistas brasileiros e apontam para a necessidade de estudos futuros.

## Coinvestigadores

Alberto de Almeida Las Casas Júnior (Goiás), Alexandre Jorge de Andrade Negri (Paraíba), Andrés Gustavo Sánchez Esteva (Tocantins), Antônio Carlos Avanza Junior (Espírito Santo), Christiano Henrique Souza Pereira (Mato Grosso do Sul), Claudine Maria Alves Feio (Pará), Daniela Martins Lessa Barreto (Alagoas), Diana Patrícia Lamprea Sepúlveda (Pernambuco), Érika Maria Gonçalves Campana (Rio de Janeiro), Evandro Guimarães de Souza (Minas Gerais), Ezilaine Nascimento Rosa (Mato Grosso), Fátima Elizabeth Fonseca de Oliveira Negri (Paraíba), Harry Corrêa Filho (Santa Catarina), João Paulo Fernandes Caixeta (Goiás), João Roberto Gemelli (Rondônia), Joilma Silva Prazeres Tobias (Maranhão), José Fernando Vilela Martin (São Paulo), Juan Carlos Yugar Toledo (São Paulo), Lara Araújo Dias (Goiás), Maurício Pimentel (Rio Grande do Sul), Nivaldo Menezes Filgueiras Filho (Bahia), Sandra Andrade Mendonça Hilgemberg (Rio Grande do Norte), Sílvio Henrique Barberato (Paraná), Simone Nascimento dos Santos (Distrito Federal), Thaynara de Moraes Pacheco (Goiás).
