# Supplementary material for: Fatores de Risco Cardiovascular em Cardiologistas Especialistas pela Sociedade Brasileira de Cardiologia
Source: Arq Bras Cardiol. 2021 Apr 8;116(4):774–81. [Article in Portuguese] doi: 10.36660/abc.20200125 (PMC8121412; doi:10.36660/abc.20200125)
Supplement: Translation

# Translation: Cardiovascular Risk Factors in Cardiology Specialists from the Brazilian Society of Cardiology

**Authors:** Maria Emília Figueiredo Teixeira, Priscila Valverde de O. Vitorino, Celso Amodeo, Tânia Martinez, Andréa Araujo Brandão, Eduardo Costa Duarte Barbosa, Audes Diógenes Magalhães Feitosa, Paulo Cesar B. Veiga Jardim, Ana Luiza Lima Souza, Weimar Kunz Sebba Barroso

**Affiliations:** 1 GoiâniaGO Brazil Universidade Federal de Goiás - Liga de Hipertensão Arterial, Goiânia, GO – Brazil.; 2 GoiâniaGO Brazil Universidade Federal de Goiás - Programa de Pós-Graduação em Ciências da Saúde, Goiânia, GO - Brazil.; 3 GoiâniaGoiás Brazil Pontifícia Universidade Católica de Goiás - Escola de Ciências Sociais e da Saúde, Goiânia, Goiás - Brazil.; 4 São PauloSP Brazil Escola paulista de Medicina da Universidade Federal de São Paulo (Unifesp EPM), São Paulo, SP - Brazil.; 5 São PauloSP Brazil Hospital do Coração (HCor) – Lipids, São Paulo, SP - Brazil.; 6 Rio de JaneiroRJ Brazil Universidade do Estado do Rio de Janeiro (UERJ) – Cardiologia, Rio de Janeiro, RJ - Brazil.; 7 Porto AlegreRS Brazil Instituto de Cardiologia - Laboratório de Investigação Clínica (LIC), Porto Alegre, RS - Brazil.; 8 RecifePE Brazil Laboratório de Imunopatologia Keizo Asami - Universidade Federal de Pernambuco - Clínica Médica, Recife, PE - Brazil.; 9 GoiâniaGO Brazil Universidade Federal de Goiás – Cardiologia, Goiânia, GO – Brazil.

**Keywords:** Cardiovascular Diseases, Cardiologists, Risk Factors, Antropometry, Hypertension, Dsylipidemias, Diabetes Mellitus, Life Style

## Abstract

**Background:**

A major cause of death worldwide, cardiovascular diseases and their prevalence in cardiologists are little known.

**Objectives:**

To describe life habits and cardiovascular risk factors (CVRF) and to investigate the prevalence of diagnosis, awareness, and control of these CVRF among cardiologists members affiliated to and specialists from the Brazilian Society of Cardiology.

**Methods:**

National multicenter cross-sectional study to assess Brazilian cardiologists using a questionnaire on life habits, preexisting diseases, current medications, anthropometric measurements, blood pressure, and levels of glucose and lipids.

**Results:**

A total of 555 cardiologists were evaluated, of which 67.9% were male, with a mean age of 47.2±11.7 years. Most were non-smoker (88.7%) and physically active (77.1%), consumed alcohol (78.2%), had normal weight circumference (51.7%), and were overweight (56.1%). The prevalence of systemic arterial hypertension (SAH), diabetes mellitus (DM), and dyslipidemia (DLP) were 32.4%, 5.9%, and 49.7%, respectively, of which only 57.2%, 45.5%, and 49.6%, respectively, were aware of the diseases.

**Conclusions:**

The Brazilian cardiologists participating in the study had a high prevalence of SAH, DM and DLP, but only a half of participants were aware of these conditions and, among these, the rates of controlled disease were low for SAH and DLP, although cardiologists are professionals with great knowledge about these CVRF. These findings represent a warning sign for the approach of CVRF in Brazilian cardiologists and encourage the conduction of future studies.

## Introduction

Among cardiovascular risk factors (CVRF), systemic arterial hypertension (SAH), diabetes mellitus (DM), dyslipidemias (DLP), and smoking are the ones with the greatest impact on increased morbidity and mortality rates.^1^ Furthermore, unfavorable life habits lead to overweight and, when combined, interfere significantly with the prevalence of CVRF,^2^ with a consequent increase in the incidence of cardiovascular outcomes, such as sudden death, stroke, acute myocardial infarction (AMI), heart failure, peripheral artery disease, and chronic kidney disease.^3^^–^^5^

Health care professionals, including physicians, especially cardiologists, play a crucial role in diagnosing and treating cardiovascular diseases.^6^ Additionally, Brazilian cardiologists are often perceived as the responsible for the overall health care of adult patients.^7^ Therefore, cardiologists are expected, in addition to providing care, to serve as a role model and, particularly, to personally engage in healthy life habits.^8^

There are few studies assessing cardiovascular risk and life habits of Brazilian cardiologists;^9^ thus, this study aimed to: (1) investigate life habits and CVRF and (2) identify the prevalence of diagnosed, self-reported, and controlled SAH, DM, and DLP in cardiologists affiliated to and specialists from the Brazilian Society of Cardiology (Sociedade Brasileira de Cardiologia, SBC).

## Methods

### Type of study, population, sample, and inclusion criteria

#### National, descriptive, cross-sectional, multicenter study.

In 2017, Brazil had 451,777 physicians, with approximately 25,000 (5.5%) cardiologists;^1^; of these, 11,495 had a cardiology specialist degree (CSD).^11^ The reference population consisted of 14,201 cardiologists members of the SBC from across the country in 2017, with state societies in 24 federative units. The research was conducted with cardiologists having CSD/SBC in an attempt to standardize the sample with regard to level of scientific knowledge.

The sample was selected by convenience and included 555 physicians with CSD/SBC and active members of SBC, which accounts for 4.8% of the reference population.

### Sites of study execution and coordination

All 24 regional representatives of SBC/Board of Cardiovascular Health Prevention (FUNCOR) were invited to participate in the group of researchers working in this project. Of these, 15 accepted the invitation and, together with three other invited centers [Instituto Dante Pazzanese de Cardiologia (IDPC), Liga de Hipertensão Arterial da Universidade Federal de Goiás (LHA/UFG), and Unidade de Hipertensão da Universidade Estadual do Rio de Janeiro], totaled 18 research centers that were effectively included in the group of investigators and coinvestigators who collected data from May to October 2017.

Data collection was conducted in the following states: Bahia, Distrito Federal, Goiás, Mato Grosso, Mato Grosso do Sul, Minas Gerais, Pará, Paraíba, Paraná, Pernambuco, Rio de Janeiro, Rio Grande do Norte, Rio Grande do Sul, Rondônia, São Paulo, and Tocantins.

The entire work was coordinated by the Board of SBC/FUNCOR, together with the university institutions IDPC and LHA/UFG.

### Study procedures

In-person meetings with all investigators were conducted in May and June 2017 to discuss study design and data collection. After receiving training, each investigator trained his/her local team for strict compliance with study procedures. Collection was made by the very responsible researcher physician, or by other dully trained cardiologists or medical students.

Study participants were explained about the aim of the study, the data collection method, and the informed consent form (ICF), which was read and signed by all participants before the start of any study procedure.

Interviews were conducted individually in a private room at a time and place previously agreed with the participants. The interview form contained questions on personal information, life habits, and personal disease history. Moreover, anthropometric blood pressure (BP) measurements were obtained, and glucose and lipid profile tests were performed.

Age was calculated from date of birth. Sex was categorized into male and female. The life habits assessed were smoking (yes/no); consumption of alcoholic beverages (yes/no, for any amount of consumption), and physical activity practice (yes/no and weekly physical activity time, with active individuals being those who reported at least 150 minutes of physical activity per week).^12^

Anthropometric variables collected were height, weight, and waist circumference. Height was reported by participants;^13^ weight was measured using an OMRON HN-290T digital weight scale, without accessories and shoes and using light clothes.^14^

Body mass index (BMI) was calculated using the weight/height^2^ formula^15^ and classified into: underweight (< 18.5 kg/m^2^), normal weight (18.5-24.9 kg/m^2^); overweight (25-29.9 kg/m^2^); class 1 obesity (30-34.9 kg/m^2^), class 2 obesity (35 -39.9 kg/m^2^), and class 3 obesity (≥ 40 kg/m^2^).^16^

Waist circumference was measured with an inelastic measuring tape^14^ and considered high if greater than 88 cm for women and greater than 102 cm for men.^17^

BP was measured using an OMRON sphygmomanometer, model HBP 1100,^18^^–^^20^ as recommended by 7^th^ Brazilian Guidelines on Arterial Hypertension.^21^ Three BP measurements were obtained, the first measurement was excluded, and the mean of the two subsequent measurements was calculated. Based on their mean BP values, participants were classified into those with normal BP (BP ≤ 120/80 mmHg), pre-hypertension (121-139/81-89 mmHg), or stage 1 hypertension (140-159/90-99 mmHg), stage 2 hypertension (160-179/100-109 mmHg), or stage 3 hypertension (BP ≥ 180/110 mmHg).^21^

Glucose and serum lipids were measured with the On Call Plus and Mission Cholesterol devices, respectively. All test measurements were directly taken from the devices in mg/dL, except for LDL, which was calculated using the Friedewald formula.^22^

Non-fasting measurements were obtained; thus, high glucose levels were considered as ≥ 160 mg/dL;^23^ and DLP was diagnosed for those with LDL 130 mg/dL and/or triglycerides ≥ 175 mg/dL.^24^

For the diagnosis of SAH, DM, and DLP, at least one of the following criteria was considered: self-report of disease, made by the participants themselves, and/or use of anti-hypertensive drugs and/or BP ≥ 140x90 mmHg in the mean of casual measurements; use of oral hypoglycemic agents and/or insulin and/or occasional blood glucose ≥ 200 mg/dL; use of statin, fibrates, ezetimibe, and/or triglycerides ≥ 175 mg/dL, and/or LDL ≥ 130 mg/dL.

Disease awareness was assessed by physicians’ self-report. Data on the frequency of SAH, DM and DLP were compared with that obtained in Brazilian National Health Survey (Pesquisa Nacional de Saúde, PNS)^25^ e in the Surveillance System for Risk and Protective Factors for Chronic Diseases by Telephone Survey (Sistema de Vigilância de Fatores de Risco para Doenças Crônicas Não Transmissíveis por Inquérito Telefônico, VIGITEL);^26^ for this analysis, only participants’ self-report was considered (reported data).

SAH was considered controlled with systolic BP < 140 mmHg and diastolic BP < 90 mmHg, DM with glucose < 200 mg/dL, and DLP with LDL < 130 mg/dL and triglycerides < 175 mg/dL.^21^^,^^23^^,^^24^

### Statistical analysis

Data were typed on the Excel for Mac software, version 16.30, and analyzed with Stata statistical analysis software, version 14. Descriptive statistics was expressed as mean, standard deviation, and absolute and relative frequencies.

### Ethical aspects

The project was developed by the FUNCOR of the SBC, 2016/2017 term, and was approved by the Research Ethics Committee of IDPC, under number 2.016.859. All participants signed an ICF before any study procedure, in compliance with Resolution 466/2012.

## Results

A total of 555 cardiologists were assessed, with a mean age of 47.2±11.7 years, of which 159 (28.6%) were from Central-West Region of Brazil, 147 (26.5%) from the Northeast Region, 103 (18.6%) from the North Region, 103 (18.6%) from Southeast Region, and 43 (7.7%) from the South Region.

Most study participants were male, were physically active, with a mean physical activity time of 200.0±106.8 minutes per week, did not smoke, and consumed alcohol (Table 1).

**Table 1:** Sample description according to sex, lifestyle, and overall health conditions, n=555, 2017

Variable	n (%)
**Sex**	
Female	178 (32.1)
Male	377 (67.9)
**Age**	
< 40 years	183 (33.2)
≥ 40 years	368 (66.8)
**Smoking**	
Yes	03 (0.5)
No	492 (88.7)
Former smoker	60 (10.8)
**Sedentary lifestyle**	
Yes	127 (22.9)
No	428 (77.1)
**Alcohol consumption**	
Yes	434 (78.2)
No	121 (21.8)
**Abdominal circumference**	
Normal	285 (51.7)
High	266 (48.3)
**Body mass index classification**	
Non-overweight	243 (43.9)
Overweight	232 (41.9)
Obesity	79 (14.2)

According to the measurements taken during the interview, most physicians presented with BP levels into the pre-hypertension category, and glucose, LDL, and triglycerides levels within normal range (Table 2).

**Table 2:** Classification of cardiologists according to blood pressure, casual glucose, and serum lipids, 2017

Classification	n (%)
**Blood pressure (n=555)**	
Normal	204 (36.8)
Pre-hypertensive	264 (47.6)
Stage I hypertension	75 (13.5)
Stage II hypertension	08 (1.4)
Stage III hypertension	04 (0.7)
**Casual glucose (n=555)**	
Normal	548 (98.7)
High	07 (1.3)
**LDL (n=538)**	
Normal	411 (76.4)
High	127 (23.6)
**Triglycerides (n=547)**	
Normal	463 (84.6)
High	84 (15.4)

The prevalence of SAH was 32.4% of participants (n=180); of these, 57.2% (n=103) were aware of their condition, and 48.3% (n=87) had their BP controlled. The prevalence of DM was 5.9% (n=33) of participants; of these, 45.5% (n=15) were aware of their condition, and 78.8% (n=26) had their glucose levels within normal range. DLP showed rates of prevalence, awareness, and control of 49.7% (n=276), 49.6% (n=137), and 31.1% (n=86), respectively (Figure 1).

**Figure 1:** Prevalence of diagnosis, awareness, and control of SAH, DM and DLP in cardiologists, n=555, 2017. DLP: dyslipidemia; DM: diabetes mellitus; SAH: systemic arterial hypertension.

With regard to cardiovascular outcomes, 4 (0.72%) cardiologists reported to have suffered an AMI, and 1 (0.18%) reported to have suffered a stroke. All four physicians with diagnosed coronary artery disease were on antiplatelet therapy.

Table 3 shows the frequencies of CVRF and cardiovascular outcomes of PNS,^25^ VIGITEL,^26^ and findings from the present study, considering only self-reported diseases.

**Table 3:** Prevalence of risk factors and cardiovascular outcomes in the general population and among cardiologists. n = 555, 2017

	PNS	VIGITEL	Cardiologists (reported)	Cardiologists (measured)
Sedentary lifestyle	46	61.9	22.9	-
Alcohol consumption	24	17.9	78.2	-
Smoking	15	9.3	0.5	-
Arterial hypertension	21,4	24.7	18.6	32.4
Diabetes mellitus	6,2	7.7	2.7	5.9
Dyslipidemia	12,5	-	24.7	49.7
Acute myocardial infarction	4,2	-	0.7	-
Stroke	1,5	-	0.2	-

Source: PNS^25^, VIGITEL 2018^26^

## Discussion

This is the first Brazilian study to assess cardiologists with CSD from the five geographical regions for the presence of CVRF and life habits. These cardiologists showed a very low prevalence of sedentary lifestyle and smoking, and a higher prevalence of alcohol consumption compared with studies that assessed the general population, such as PNS^25^ and VIGITEL,^26^ as well as a higher prevalence of DLP, a slightly lower prevalence of SAH, and a lower prevalence of DM. However, the rates of awareness of SAH, DM and DLP and the rates of control of SAH and DLP were low, considering that the study population consisted of cardiologists, which are supposed to understand the importance of controlling CVRF.

In the Brazilian population, the prevalence of SAH ranges from 30% to 36%;^27^^,^^28^ the prevalence of DM is 11.4%;^29^ and the prevalence of DLP is divided into hypercholesterolemia, with a prevalence of approximately 45.5%,^30^ and hypertriglyceridemia, with a prevalence from 26.5% to 31. 2% in Latin America.^31^^,^^32^ Furthermore, the prevalence of excess weight (overweight/obesity) in Brazil is 57% in men and 43% in women.^33^ In the present study group, considering reported and measured data, the diagnosis rate was 32.4% for SAH, 4.9% for DM, 51.7% for DLP (hypercholesterolemia and/or hypertriglyceridemia), and 56% for excess weight (67.1% in men and 32.2% in women).

Lack of awareness of these CVRF is known to be high in the general population, but strikingly, it is also high among cardiologists, which lead us to consider that these professionals neglect their own health care. This delay in disease awareness, early diagnosis, and appropriate treatment may increased the risk of related outcomes.^34^

Health education to the lay population is knowingly able to improve live habits, leading to a decrease in cardiovascular diseases.^35^ Hence, there was the questioning on the quality of cardiologists’ self-care, since they are the bearers of this scientific knowledge. Medical students assessed for CVRF had a similar prevalence than that of the general population of the same age, except for higher rates of sedentary lifestyle and higher BMI, thus raising a discussion on the extensive workload of the course, which may influence on the low time availability for the practice of healthy life habits, compared with other young adults.^36^ In another group of medical students, obesity rates were lower compared with those of population of the same age, as well as better serum lipid levels, but they showed high consumption of fast food and alcohol, in addition to higher rates of sedentary lifestyle, which may also be explained by low time availability and the high level of stress related to the course.^37^

It is known that work routine may often have a negative impact on the adoption of health and wellbeing practices, even if the professional have knowledge on the theme, such as health care professionals.^38^ The work in this area requires working in night shifts, and professionals often have more than one job. Therefore, they have difficulty in practicing regular physical activity or prioritizing nutritionally balanced meals.

Conversely, the same discussion may be raised without the need of emphasizing the night shift as the most important harm, but considering only the excessive workload of these professionals, regardless of the period of the day. Two different groups assessed their professionals with regard to the prevalence of CVRF, including the entire multiprofessional team in the assessment. In a general hospital, a high prevalence of CVRF were observed in all assessed professional categories.^39^ Similar results were found in another group, with an even more worrisome situation, which is the lack of awareness of these individuals with regard to their already altered health satus.^40^

In the subgroups of cardiologists versus non-cardiologists physicians, no significant differences were observed in relation to serum levels of cholesterol and its fractions, as well as to Framingham risk score, but cardiologists consumed more alcohol, and both groups had a mean BMI above the ideal range.^41^

In a comparative analysis with the population surveys PNS^25^ and VIGITEL,^26^ the cardiologists assessed in the present study reported lower rates of smoking and sedentary lifestyle, but come more alcohol. Furthermore, considering only reported CVRF, cardiologists reported lower rates of SAH and DM, but higher rates of DLP. These data are worrisome, not only due to lack of awareness, but also because they call into question the credibility of surveys that use only reported data.

SAH, DM and DLP^42^ are known to result from factors such as genetics and aging (non-modifiable), but are also related to life habits, and, within this context, individual with greater knowledge on cardiovascular risk factors are expected to have healthier habits.^43^^–^^45^ With wide knowledge on the topic, cardiologists were expected to fully engage in good habits, so as to prevent these diseases, which is contrary to the findings in our sample with regard to alcohol consumption, but is consistent with findings related to smoking and physical activity. Similarly, a similar, or even higher, prevalence was found for the main CVRF, in comparison to the general population, except for DM.

Finally, the percentage of reported AMI (0.72%) and stroke (0.18%) in the sample was much lower than that of the general population, which may be related to the regular and frequent use of medications, due to physicians’ knowledge on the appropriate treatment and ease of access to medications. Furthermore, mean age of the group was low (47.2 years) and may partly justify the low prevalence of AMI and stroke.^46^

The present study had the following limitations: the lack of HDL in the assessment for DLP, due to a limitation in the measuring device; lack of administration of instruments to assess physical activity and alcohol consumption, which may have overestimated these rates; and the fact that fasting biochemical tests were not obtained. Nonetheless, it is worth noting that equal devices were used to obtain both anthropometric measurements and BP value and blood biochemistry tests, with previous training of coinvestigators and general coordination of reference centers, showing an appropriate standardization of the procedure.

It is also worth emphasizing that the sample was not representative of cardiologists affiliated to the SBC, because this was a convenience sample, a fact that may relativize the results and the presented discussions. However, cardiologist from all over the country were assessed and, thus, this study represents a warning sign for the approach of the identified conditions and for the conduction of future studies with Brazilian cardiologists.

## Conclusion

Most cardiologists were male, were physically active, did not smoke, consumed alcohol, and had a significant prevalence of SAH, DM and DLP, similar to those observed in other surveys with Brazilian populations. However, although cardiologist have knowledge on these CVRF, approximately a half of them were aware of these conditions and were with their pressure controlled; additionally, one third had their lipid levels within normal values, but most had their glucose levels controlled. Study findings represent a warning sign for the adequate approach of CVRF among Brazilian cardiologists and point to the need of future studies.

## Coinvestigators

Alberto de Almeida Las Casas Júnior (Goiás), Alexandre Jorge de Andrade Negri (Paraíba), Andrés Gustavo Sánchez Esteva (Tocantins), Antônio Carlos Avanza Junior (Espírito Santo), Christiano Henrique Souza Pereira (Mato Grosso do Sul), Claudine Maria Alves Feio (Pará), Daniela Martins Lessa Barreto (Alagoas), Diana Patrícia Lamprea Sepúlveda (Pernambuco), Érika Maria Gonçalves Campana (Rio de Janeiro), Evandro Guimarães de Souza (Minas Gerais), Ezilaine Nascimento Rosa (Mato Grosso), Fátima Elizabeth Fonseca de Oliveira Negri (Paraíba), Harry Corrêa Filho (Santa Catarina), João Paulo Fernandes Caixeta (Goiás), João Roberto Gemelli (Rondônia), Joilma Silva Prazeres Tobias (Maranhão), José Fernando Vilela Martin (São Paulo), Juan Carlos Yugar Toledo (São Paulo), Lara Araújo Dias (Goiás), Maurício Pimentel (Rio Grande do Sul), Nivaldo Menezes Filgueiras Filho (Bahia), Sandra Andrade Mendonça Hilgemberg (Rio Grande do Norte), Sílvio Henrique Barberato (Paraná), Simone Nascimento dos Santos (Distrito Federal), Thaynara de Moraes Pacheco (Goiás).
